# Sex, Age, and Socioeconomic Differences in Nonfatal Stroke Incidence and Subsequent Major Adverse Outcomes

**DOI:** 10.1161/STROKEAHA.120.031659

**Published:** 2021-01-26

**Authors:** Ralph K. Akyea, Yana Vinogradova, Nadeem Qureshi, Riyaz S. Patel, Evangelos Kontopantelis, George Ntaios, Folkert W. Asselbergs, Joe Kai, Stephen F. Weng

**Affiliations:** 1 Primary Care Stratified Medicine, Division of Primary Care, University of Nottingham, Nottingham, United Kingdom (R.K.A., Y.V., N.Q., J.K., S.F.W.).; 2 Institute of Cardiovascular Science, Faculty of Population Health Sciences (R.S.P., F.W.A.), University College London.; 3 Health Data Research UK, Institute of Health Informatics (R.S.P., F.W.A.), University College London.; 4 Division of Population Health, Health Services Research and Primary Care (E.K.), School of Health Sciences, Faculty of Biology, Medicine and Health, Manchester Academic Health Science Centre (MAHSC), The University of Manchester, United Kingdom.; 5 Division of Informatics, Imaging, and Data Sciences (E.K.), School of Health Sciences, Faculty of Biology, Medicine and Health, Manchester Academic Health Science Centre (MAHSC), The University of Manchester, United Kingdom.; 6 Department of Internal Medicine, Faculty of Medicine, School of Health Sciences, University of Thessaly, Larissa, Greece (G.N.).; 7 Division Heart and Lungs, Department of Cardiology, University Medical Center Utrecht, Utrecht University, the Netherlands (F.W.A.).

**Keywords:** cardiovascular diseases, epidemiology, incidence, population, secondary prevention

## Abstract

Data about variations in stroke incidence and subsequent major adverse outcomes are essential to inform secondary prevention and prioritizing resources to those at the greatest risk of major adverse end points. We aimed to describe the age, sex, and socioeconomic differences in the rates of first nonfatal stroke and subsequent major adverse outcomes.

**Methods:**

The cohort study used linked Clinical Practice Research Datalink and Hospital Episode Statistics data from the United Kingdom. The incidence rate (IR) ratio of first nonfatal stroke and subsequent major adverse outcomes (composite major adverse cardiovascular events, recurrent stroke, cardiovascular disease-related, and all-cause mortality) were calculated and presented by year, sex, age group, and socioeconomic status based on an individual’s location of residence, in adults with incident nonfatal stroke diagnosis between 1998 and 2017.

**Results:**

A total of 82 774 first nonfatal stroke events were recorded in either primary care or hospital data—an IR of 109.20 per 100 000 person-years (95% CI, 108.46–109.95). Incidence was significantly higher in women compared with men (IR ratio, 1.13 [95% CI, 1.12–1.15]; *P*<0.001). Rates adjusted for age and sex were higher in the lowest compared with the highest socioeconomic status group (IR ratio, 1.10 [95% CI, 1.08–1.13]; *P*<0.001). For subsequent major adverse outcomes, the overall incidence for major adverse cardiovascular event was 38.05 per 100 person-years (95% CI, 37.71–38.39) with a slightly higher incidence in women compared with men (38.42 versus 37.62; IR ratio, 1.02 [95% CI, 1.00–1.04]; *P*=0.0229). Age and socioeconomic status largely accounted for the observed higher incidence of adverse outcomes in women.

**Conclusions:**

In the United Kingdom, incidence of initial stroke and subsequent major adverse outcomes are higher in women, older populations, and people living in socially deprived areas.

## Abstract 2

Supplemental Digital Content is available in the text.

Stroke is the second leading cause of death globally^1^ and a leading cause of disability worldwide.^2^ The aging population and treatment improvements are expected to significantly impact on stroke epidemiology^3^—the number of stroke survivors is on the rise, impacting the need for poststroke facilities and the risk of stroke recurrence.^4^ About 1 in 4 stroke survivors will experience another stroke within 5 years.^5^ Despite advances in the management of patients with stroke, mortality, and disability rates remain high.^6^

There is a lack of contemporary evidence from nationally representative data across the entire spectrum of both primary and secondary care to assess demographic variations in the incidence of first nonfatal stroke. Most published estimates of stroke incidence in the United Kingdom only capture certain types of stroke, may or may not include recurrent stroke, focus on small nonrepresentative populations or a short time period.^7–9^ Moreover, the sex and age variations for the incidence of major adverse outcomes after the first stroke have either been in selected populations with incident ischemic stroke and above a predefined age.^10,11^ To identify patterns and any discrepancies in care of patients, information on temporal trends in sex- and age-related differences with respect to the incidence of stroke and subsequent major adverse outcomes is needed. This can inform resource allocation and policy to enhance clinical care and outcomes.

We sought to update current knowledge on differences in nonfatal stroke incidence and incidence of major adverse outcomes following the first nonfatal stroke event. We used linked electronic health record from primary care consultations, secondary care (hospital admissions and procedure-level data), and the national death registry that are representative of the UK population. This population-based study explores demographic variations in incidence of stroke (first-ever nonfatal stroke) and incidence of major adverse outcomes after first-ever stroke among individuals aged 18 years and over.

## Methods

### Data Availability

The data that support the findings of this study are available from Clinical Practice Research Datalink (CPRD). Restrictions apply to the availability of the data, which was used under license for the current study, and so are not publicly available. However, data is available upon application to CPRD.

### Data Source

This prospective cohort study used the UK CPRD GOLD of primary care electronic health records,^12^ linked to Hospital Episode Statistics (HES),^13^ Office for National Statistics mortality data,^14^ and social deprivation data.^15^ CPRD collects deidentified patient data (including diagnoses, symptoms, prescriptions, referrals, and tests) from data quality-assured GP practices across the United Kingdom.^12^ Patients in CPRD are broadly representative of the UK general population in terms of sex, age, and ethnicity.^12,16^ CPRD is very widely used internationally for epidemiological research and has been used to produce over 1000 research studies, published in peer-reviewed journals across a broad range of health outcomes.^12^ HES is a national database containing dates and diagnostic codes for all elective and emergency admissions and procedures to National Health Service hospitals in England (https://www.hscic.gov.uk/hes). Office for National Statistics provides data on cause-specific mortality and corresponding dates obtained from death certificates for all residents in the United Kingdom.^14^ This study was approved by the Independent Scientific Advisory Committee of the Medicines and Health care products Regulatory Agency (Protocol number 19_023R).

### Study Population

We identified a cohort of patients with first record of nonfatal stroke in either CPRD GOLD or HES data between January 1, 1998, and December 31, 2018. Patients entered the study cohort at minimum date of study start (January 1, 1998); being aged 18 years and over; with at least 12 months of registration^17^; practice data of acceptable quality (up-to-standard—Method in the Data Supplement); and eligible for linkage to HES. The follow-up end date was defined as the date of transfer out/leaving the practice, date of death, last date for CPRD-HES link, or the last date of data collection, whichever came earliest. Patients with a prior history of stroke before the study start date (January 1, 1998) were excluded from the study—5621 records between 1914 and 1997.

To assess the incidence of subsequent major adverse outcomes, the cohort consisted of patients with no prior history of major adverse event (ie, coronary heart disease [CHD], including coronary revascularization, peripheral vascular disease [PVD], or heart failure). Patients were followed until they developed a major adverse outcome or left the original cohort. Figure I in the Data Supplement shows the study flow diagram.

### Identifying Patients, Patient Characteristics, and Outcomes

To identify patients with stroke and outcomes of interest, we adapted the code list from the CALIBER code repository^18^ (https://www.caliberresearch.org/portal/)—from CPRD GOLD using Read codes, from HES using *International Classification of Diseases, Tenth Revision* codes and Office of Population Censuses and Surveys Classification of Surgical Operations and Procedures revision 4.6 for procedure codes—Table I in the Data Supplement. We also extracted information on demographic factors, including age and sex, socioeconomic status (SES), and ethnicity. For SES, a key determinant for health, we used the English Index of Multiple Deprivation (IMD) 2015^15^ which is a widely used composite measure of deprivation. The current IMD measure quantifies relative deprivation across 7 different domains of deprivation: income; employment; education, skills, and training; health and disability; crime; barriers to housing and services; and living environment, using an area-based model at a low geography of about 1500 people.^19^ The overall IMD is calculated as a weighted mean across the 7 domains, hence, offers a single score to describe the concept of deprivation whiles recognizing the many interacting components. SES is ranked into quintiles (quintile 1: highest SES group to quintile 5: lowest SES group).

The outcomes of interest were subsequent major adverse events (composite major adverse cardiovascular events [MACE], recurrent stroke, cardiovascular disease [CVD]-related, and all-cause mortality) after incident stroke. MACE was defined as a composite of CHD, stroke, PVD, heart failure, and CVD-related death) based on records from CPRD GOLD, HES, or Office for National Statistics registries.

### Statistical Analysis

Baseline characteristics were presented by sex and expressed using mean and SD for continuous variables (after assessment of the normality of data) and percentages for categorical variables. Differences for categorical and continuous variables were assessed using the χ^2^ and *t* tests, respectively. Incidence rates (IRs) per 100 000 person-years for stroke were calculated by dividing the number of individuals with a stroke by the number of person-years of all patient in the original cohort. IRs were presented by age (5-year intervals), sex, and year of diagnosis. IR ratios (IRRs) for IMD quintiles were adjusted for age and sex in a Poisson regression model.

We calculated the IRs per 100 person-years for subsequent major adverse outcomes in the cohort of patients with no prior history of major adverse event. A sensitivity analysis was restricted to the cohort of patients with subsequent major adverse outcomes occurring after 30 days of the index stroke was done. Patients with other associated major adverse end points are more likely to remain in hospital for an extended period following admission for their incident stroke event. The recording of such hospital (secondary care) activity in primary care records through discharge letters or referral notes from specialists may be delayed. The date of referral/letter may erroneously be recorded as the date of stroke or associate major adverse outcome. Some outcome events may only be recorded when a posthospitalization visit to primary care occurs. Hence, a 30-day interval was chosen because records for adverse outcomes occurring within 30 days is likely to be related to index stroke event.^20,21^

The study findings are reported in accordance with the Reporting of Studies Conducted Using Observational Routinely Collected Health Data recommendations.^22^ All statistical analyses were performed using Stata 16.1 (StataCorp LP), and statistical significance was set at *P*<0.05.

## Results

A total of 9 992 380 individuals 18 years and over were identified in CPRD GOLD with a total follow-up time of 75 794 468.8 person-years between January 1, 1998, and December 31, 2017. The mean follow-up time was 1.81 years (SD: 2.78) with a median of 0.51 years (interquartile range, 0.05–2.41). There were 82 774 individuals with incident nonfatal stroke. There were 44 614 (53.9%) women. The mean age at incident stroke was 74.3 year (SD: 13.6). Males had incident stroke at a younger age compared with women (71.4 versus 76.9 years). Hypertension was the most prevalent comorbid condition (48.4%)—see Table 1 for details.

**Table 1.**
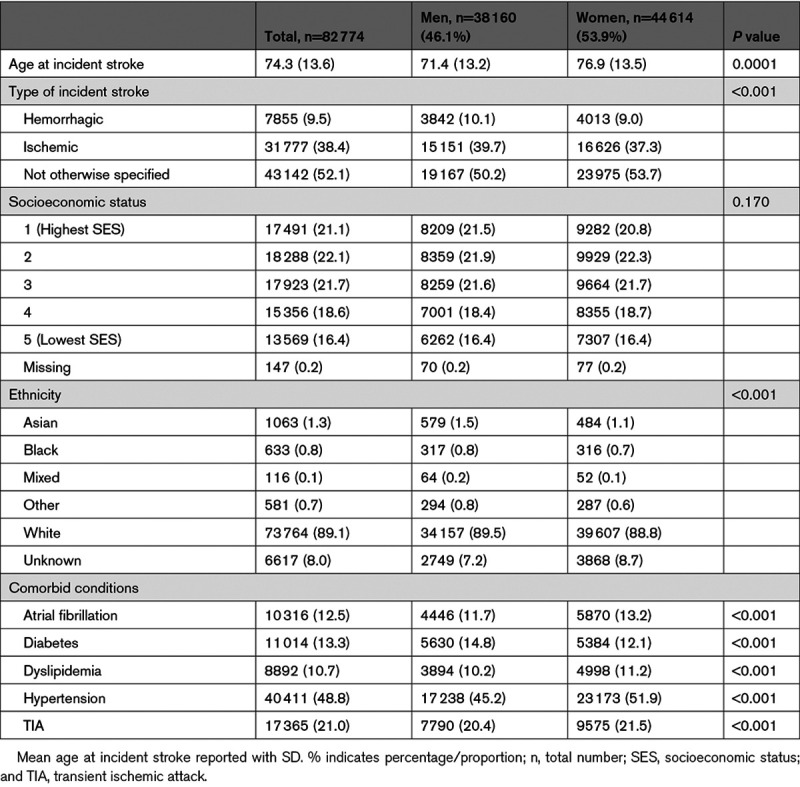
Demographic Characteristics of Individuals Aged 18 Years or Above With Incident Nonfatal Stroke, n=82 774

### Overall Stroke Incidence

**Figure 1.**
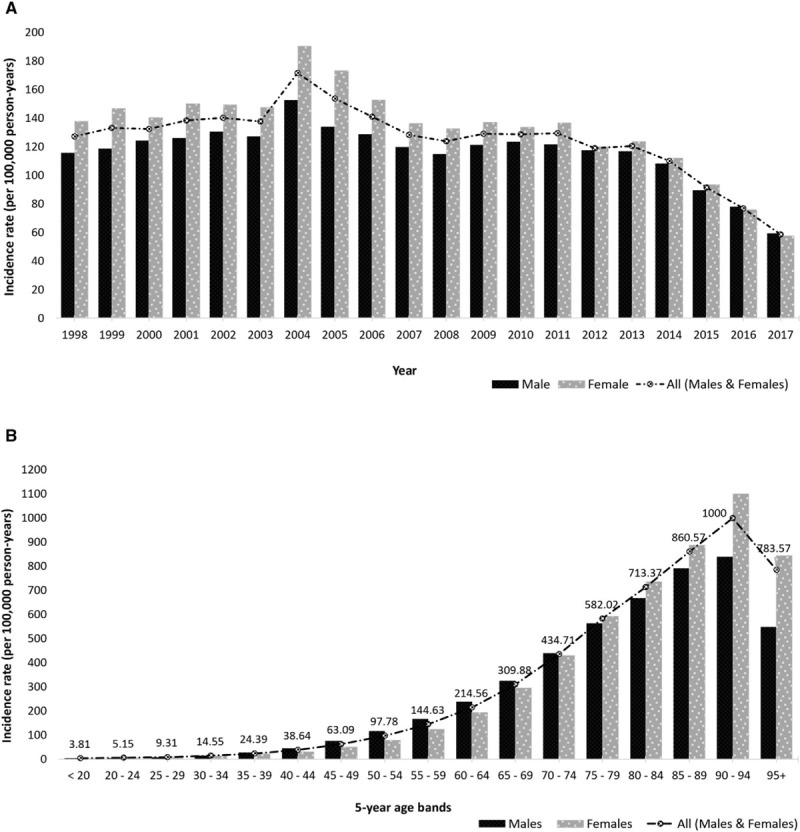
**Trends in stroke incidence.** **A**, Trend in stroke incidence presented by sex (1998–2017). **B**, Trend of stroke incidence presented by age group and sex (1998–2017).

The overall IR of stroke from 1998 to 2017 was 109.21 per 100 000 person-years (95% CI, 108.47–109.96). The IR was relatively steady between 1998 and 2003, with a peak incidence in 2004, and a subsequent decline in incidence till 2017, as shown in Figure 1A (details in Table II in the Data Supplement). The overall stroke incidence was higher in women (IR, 115.84 [95% CI, 114.77–116.92]) compared with males (IR, 102.36 [95% CI, 101.34–103.39]) with an IRR of 1.13 (95% CI, 1.12–1.15, *P*<0.0001). Stroke incidence increased in age groups older than the 55- to 59-year group—Figure 1B shows variations and Table III in the Data Supplement provides the detailed results. Males aged 30 to 74 years had higher stroke IRs compared with women, however, from age 75 the IRs were much higher in women.

### Stroke Incidence by SES

After adjusting for the effects of age and sex (Table IV in the Data Supplement), we observed increasing incidence for every increase in IMD quintile. The rate of stroke incidence among individuals in the lowest SES quintile was 10% higher than the rate in the highest SES quintile (IRR, 1.10 [95% CI, 1.08–1.13]).

### Subsequent Major Adverse Outcomes

**Figure 2.**
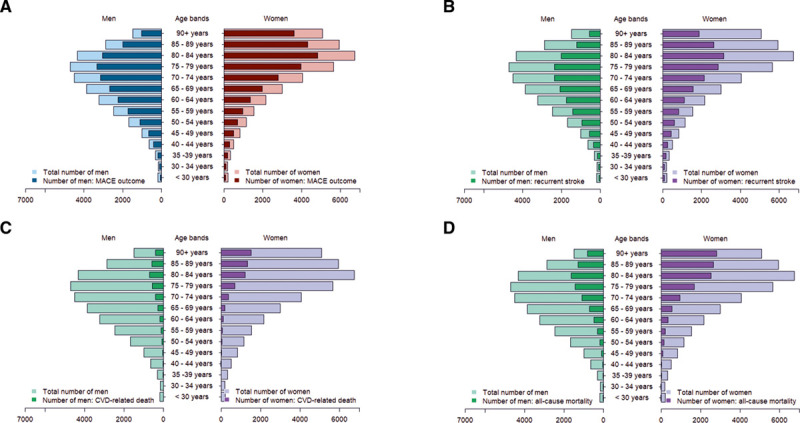
**Distribution of subsequent major adverse outcomes presented by sex and 5-y age groups (n=68 877).** **A**, Major adverse cardiovascular event (n=47 500), (**B**) recurrent stroke (n=33 831), (**C**) cardiovascular-related mortality (n=9174), and (**D**) all-cause mortality (n=20 335).

Of the 82 774 individuals with incident stroke event, 13 897 had a prior history of major adverse outcome and were excluded. Of the 68 877 individuals, mean age at incident stroke was 73.3 year (SD: 13.9), with 37 395 (54.3%) being women. With respect to outcomes, 47 500 (69.0%) had a MACE; 33 831 (49.1%) recurrent stroke (hemorrhagic stroke: 2378 [4.1%], ischemic stroke: 8842 [15.1%], stroke [not specified]: 22 611 [38.6]); 9174 (13.3%) cardiovascular death; and 20 335 (29.5%) all-cause death, occurring after the incident stroke events—Table V in the Data Supplement. There were 25 731 (68.8%) women with MACE outcome. Figure 2 shows the distribution of MACE, recurrent stroke, cardiovascular, and all-cause mortality outcomes presented by sex and across 5-year age bands. Most subsequent outcomes occurred within 2 years of incident stroke—with the median follow-up time at which outcomes occurred after incident stroke ranging between 0.10 years (interquartile range, 0.02–1.49) for CVD-related mortality and 1.74 years (interquartile range, 0.51–4.42) for heart failure.

### Incidence of Subsequent Major Adverse Outcomes

**Figure 3.**
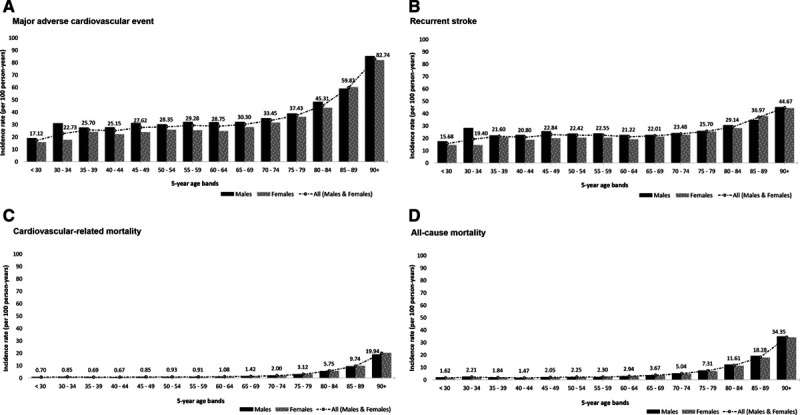
**Incidence of subsequent major adverse outcomes presented by sex and 5-y age groups (n=68 877).**

The overall MACE IR was 38.05 per 100 person-years (95% CI, 37.71–38.39). There was a steady rise in MACE incidence across the various age groups before peaking in the 80+ age group with a MACE IR of 45.31 per 100 person-years as illustrated in Figure 3. With respect to the constituent MACE outcomes, women had a higher IR for CVD-related (4.13 versus 2.72 per 100 person-years respectively; IRR, 1.52 [1.45–1.58]) and all-cause mortality (8.45 versus 6.21 per 100 person-years, respectively; IRR, 1.36 [1.32–1.40]). The incidence of CHD and PVD were, however, higher in men. Table 2 details the sex variation in the incidence of the constituent MACE outcomes. In comparing women to men, the age- and SES-adjusted sex-specific IRR for MACE was 0.92 (0.90–0.94), recurrent stroke: 0.96 (0.94–0.98), CVD-related death: 1.00 (0.96–1.05), all-cause mortality: 0.96 (0.93–0.98), CHD: 0.75 (0.69–0.81), PVD: 0.64 (0.54–0.76), and heart failure: 0.93 (0.84–1.03).

**Table 2.**
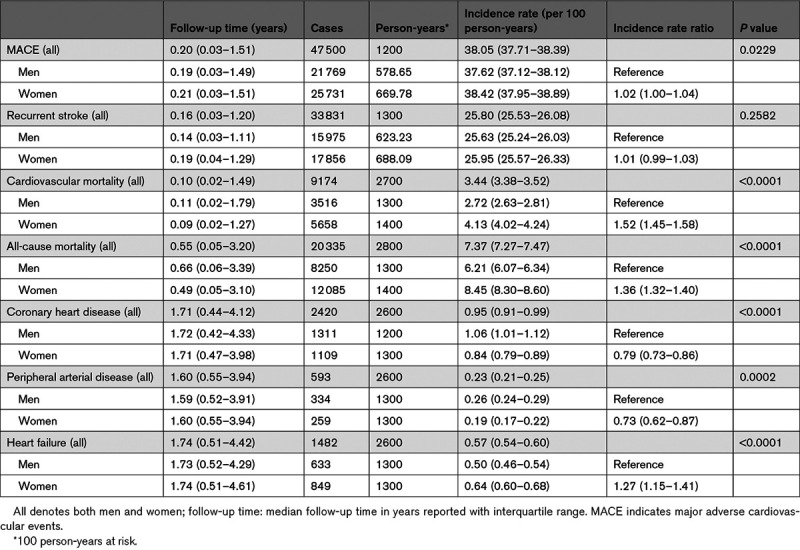
Incidence of Subsequent Major Adverse Outcomes (n=68 877)

### Subsequent Major Adverse Outcomes by SES

After adjusting for age and sex, the rate ratio of MACE incidence among individuals in the lowest SES quintile was 9% more than the rate in the highest SES quintile (IRR, 1.09 [95% CI, 1.06–1.13]). There was no significant difference in recurrent stroke incidence between individuals in the lowest and highest SES quintiles, IRR 1.00 (95% CI, 0.97–1.04)—Table VI in the Data Supplement.

### Sensitivity Analysis

For the sensitivity analysis, 20 571 (29.9%) of the 68 877 patients with subsequent major adverse outcome with 30 days of incident stroke were excluded. The remaining follow-up cohort (n=48 306) had similar strata for SES as the excluded cohort. The proportion of patients with prestroke comorbid conditions varied between the excluded and remaining cohorts: atrial fibrillation (10.9% versus 8.7%, *P*<0.001), dyslipidemia (8.3% versus 10.0%, *P*<0.001), transient ischemia attack (8.2% versus 25.6%, *P*<0.001) respectively—Table VII in the Data Supplement. There was a total of 28 750 (59.5%) subsequent MACE outcomes recorded. Figure II and Table VIII in the Data Supplement show the distribution of subsequent MACE and its constituent outcomes: recurrent stroke (n=19 896, 41.2%), cardiovascular mortality (n=4797, 9.9%), and all-cause mortality (n=14 137, 29.3%). The overall IR for subsequent MACE was 23.14 per 100 person-years (95% CI, 22.87–23.41), lower than the rate in the main analysis cohort (38.05 [95% CI 37.71–38.39]). Similar patterns for the incidence of subsequent major outcomes by sex and across the 5-year age groups, Figure III in the Data Supplement, were obtained. However, the IRs per 100 person-years were slightly lower when compared with the main analysis—recurrent stroke (15.43 versus 25.80), cardiovascular mortality (2.34 versus 3.44), and all-cause mortality (6.58 versus 7.37), Table IX in the Data Supplement. The rate ratios by SES remained similar, Table X in the Data Supplement.

## Discussion

The incidence of stroke in the general population over the study period 1998 to 2019 was 109.20 per 100 000 person-years, with a peak incidence in 2004 and then a steady decline over the following years. The incidence of stroke was higher in women. Our findings show the greater burden of major adverse outcomes observed in women when compared with men after first nonfatal stroke is largely accounted for by age and SES. The incidence of first stroke and subsequent all-cause mortality are higher in individuals in the lowest compared with the highest SES groups.

Findings from a meta-analysis using the Global Burden of Disease analytical technique (DisMod-MR), also estimated stroke incidence in the United Kingdom to be 120 strokes per 100 000 population.^23^ A UK study with a smaller population of 1657 individuals with acute vascular events in 9 Oxford primary care practices between 2002 and 2005, reported an IR of 141 per 100 000 population (95% CI, 127–156) for ischemic stroke with women having a higher IR than men (147 versus 136, respectively).^9^ By combining both primary care and hospital data in our study, out-of-hospital stroke events are more likely to be captured,^24^ hence, more precise estimates of incidence.

The Quality and Outcome Framework, a pay-for-performance scheme covering a range of clinical and organizational areas in primary care, was introduced in the United Kingdom in April 2004.^25^ Stroke was one of the 11 areas within the clinical domain. Although the Quality and Outcome Framework is a voluntary system, 99% of UK practices participate.^25^ During the first year, the level of achievement exceeded the governments anticipated level with an average of 83.4% of the allocated incentive payments claimed.^26^ The rise in stroke incidence in 2004 could be attributed to better recording due to the introduction of the Quality and Outcome Framework. This pay scheme also incentivized primary prevention, in particular, blood pressure and cholesterol control. These might have impacted on the incidence of stroke post 2004.

In England, Wales, and Northern Ireland, a study by Wang et al,^27^ reported the average age for incident stroke to be 72 years for men and 78 for women (71.4 and 76.8 years, respectively, in our study). Consistent with other studies, women had a higher rate of major adverse outcomes in the acute phase and were less likely to survive following stroke compared with males.^28,29^ Women have more severe strokes than men,^28,29^ and the quality of care received by women with stroke has been shown to be lower than that for men.^30^ These are considered to be other possible reasons for the observed sex differences in outcome. From a public health perspective, is there is a need to monitor and compare stroke burden over time.^31^ Population-based data sets such as electronic health records offer the opportunity to explore stroke burden and its association with risk factors such as SES at a scale not previously possible.^32^ Quantifying that association between SES and stroke may help guide efforts aimed at reducing stroke burden, through local and focused secondary prevention interventions. Our study is consistent with a number of studies indicating person-level measures of lowest SES is associated with a higher risk of first-ever stroke.^33,34^ There is evidence of disparities in some aspects of stroke care and use of secondary prevention services for stroke—prescription of anticoagulation for atrial fibrillation, and timely admission to a specialist stroke unit.^34^

We were not able to identify any prior studies specifically describing the differences in IRs for subsequent major adverse outcomes after the first stroke. The closest recent studies assessing major adverse outcomes after the first stroke have been in selected populations with ischemic stroke and above a predefined age.^10,11^ Study by Sposato et al,^11^ investigating sex-specific risks (not incident rate) of incident MACE in patients ≥66 years without known CVD comorbidities with first-ever ischemic stroke and propensity-matched individuals without stroke, found no sex difference in risk of incident MACE. Most women in their cohort were likely postmenopausal hence higher testosterone/estradiol ratios or lower estrogen levels in women may have evened the risk of MACE across sexes.^35^ The higher rates of CHD and PVD in men compared with women in our study, are in keeping with previous studies.^36,37^ The risk of subsequent stroke within 90 days after acute transient ischemic attack or minor stroke has been shown to be high,^38,39^ and this could explain the high proportion transient ischemic attack recorded in individuals with a subsequent event within 30 days of incident stroke. In a study by Bray et al,^34^ patients from most deprived areas had lower 1-year survival compared with those from less deprived areas, however, the effect of SES was decreased after adjusting for baseline comorbidities. Efforts to reduce disparities in stroke and subsequent outcomes need to address not only the access to good quality health care but also the social determinants of health and vascular risk factors earlier in life.

To our knowledge, our study is the most recent largest general population study to estimate the incidence of stroke and MACE following first stroke using multiple data linkages to maximize ascertainment of stroke and MACE outcomes. The failure to use linked primary care and hospital data have been shown to lead to a substantial (25%–50%) underestimation of the burden of cardiovascular disease like acute myocardial infarction.^20^ CPRD, primary care database representative of the UK general population,^12^ is a rich source of longitudinal data and has been used to assess the incidence of a variety of health conditions.^12,40^ We, therefore, assume the incidence of stroke and subsequent major adverse outcomes within the practices contributing data to CPRD accurately reflects the incidence in the wider UK population.

Considering limitations of our study, case ascertainment is a potential limitation as the study is reliant on the presence of clinical codes indicative of stroke or any of the subsequent major adverse outcomes. Inaccurate recording may affect the estimates as is the case with all epidemiological studies using routine medical records. However, considering these conditions are Quality and Outcome Framework dependent and incentivized, the quality of the data remains high. It is not possible to be completely certain that subsequent coding of strokes does not relate to ongoing care of the initial stroke; hence, the rates for stroke maybe overestimated. Excluding patients without 12 months of data before incident stroke event minimizes the likelihood of overestimating stroke incidence in our study. Due to limited completeness of ethnicity information in people who were registered with CPRD up until 2006,^16^ and small numbers within minority ethnic groups, ethnic differences were not assessed in our study.

## Conclusions

This large population-based study linking national databases has shown that stroke remains an important public health problem. There is significant morbidity and mortality within 2 years of first stroke, with particular discrepancies across ages, sex, and SES. Public health policies and future clinical research need to investigate why this may occur, with a particular need to address disparities in outcomes for women and deprived populations. Evidence of variation in major adverse outcomes poststroke by demographic and socioeconomic characteristics offers the opportunity to tailor secondary prevention and prioritize limited health care resources to those at greatest risk.

## Acknowledgments

We thank the practices that contributed to the Clinical Practice Research Datalink (CPRD) GOLD. R.K. Akyea and Dr Weng were involved in the study conception. Analysis by R.K. Akyea with guidance from Drs Vinogradova and Weng. The article was drafted by R.K. Akyea. All authors reviewed and approved the final article. R.K. Akyea is the guarantor of the study.

## Sources of Funding

R.K. Akyea is funded by the National Institute for Health Research School for Primary Care Research (NIHR-SPCR) PhD Studentship award, supervised by Drs Weng, Qureshi, and Kai. The views expressed are those of the authors and not necessarily those of the NIHR, the NHS, or the Department of Health. Dr Patel has funding from British Heart Foundation and the National Institute for Health Research.

## Disclosures

Dr Qureshi was a member of the most recent NICE Familial Hypercholesterolemia and Lipid Modification Guideline Development Groups (CG71 and CG181) and has received honorarium from AMGEN outside the submitted work. Dr Weng reports grants from the National Institute for Health Research (PhD studentship for R.K. Akyea) during the conduct of the study; personal fees from AMGEN outside the submitted work; and is an employee of Janssen (since September 7, 2020). R.K. Akyea currently holds an NIHR-SPCR funded studentship (2018-2021). Dr Asselbergs is supported by UCL Hospitals NIHR Biomedical Research Center. The other authors report no conflicts.

## Supplemental Materials

Supplemental Methods

Figures I–III

Tables I–X

## Supplementary Material


