# Supplementary material for: Sex, Age, and Socioeconomic Differences in Nonfatal Stroke Incidence and Subsequent Major Adverse Outcomes
Source: Stroke. 2021 Jan 26;52(2):396–405. doi: 10.1161/STROKEAHA.120.031659 (PMC7834661; doi:10.1161/STROKEAHA.120.031659)
Supplement: Supplementary file 1 [file str-52-396-s001.pdf]

SUPPLEMENTAL MATERIAL

Supplemental Method Glossary of terms

Acceptable Patients

Patients are labelled as 'acceptable' for use in research by a process that identifies and excludes patients with non-continuous follow up or patients with poor data recording that raises suspicion as to the validity of the that patients record. Patient data is checked, for the following issues:

- An empty or invalid first registration date
- An empty or invalid current registration date
- Absence of a record for a year of birth
- A first registration date prior to their birth year
- A current registration date prior to their birth year
- A transferred-out reason with no transferred-out date
- A transferred-out date with no transferred-out reason
- A transferred-out date prior to their first registration date
- A transferred-out date prior to their current registration date
- A current registration date prior to their first registration date
- A gender other than Female/Male/Indeterminate
- An age of greater than 115 at end of follow up
- Recorded health care episodes in years prior to birth year
- All recorded health care episodes have empty or invalid event dates
- Registration status of temporary patients

If any of these conditions are true, the patient is labelled unacceptable and is not recommended for use in research.

UTS date

The overall quality of data in practices is mediated by use of an 'up to standard' (UTS) date, which is deemed as the date at which data in the practice is considered to have continuous high-quality data fit for use in research. This is mediated by an analysis on the total data in the practice, which is refreshed every time a new collection for a practice is processed into the database. It is based on two central concepts: assurance of continuity in data recording (gap analysis), and avoidance of use of data for which transferred out and dead patients have been removed (death recording).

The UTS date is set to the latest of these dates for each practice. The CPRD recommend that analyses are performed on data following the practice UTS date.

Supplemental Figure I Study Flow Diagram

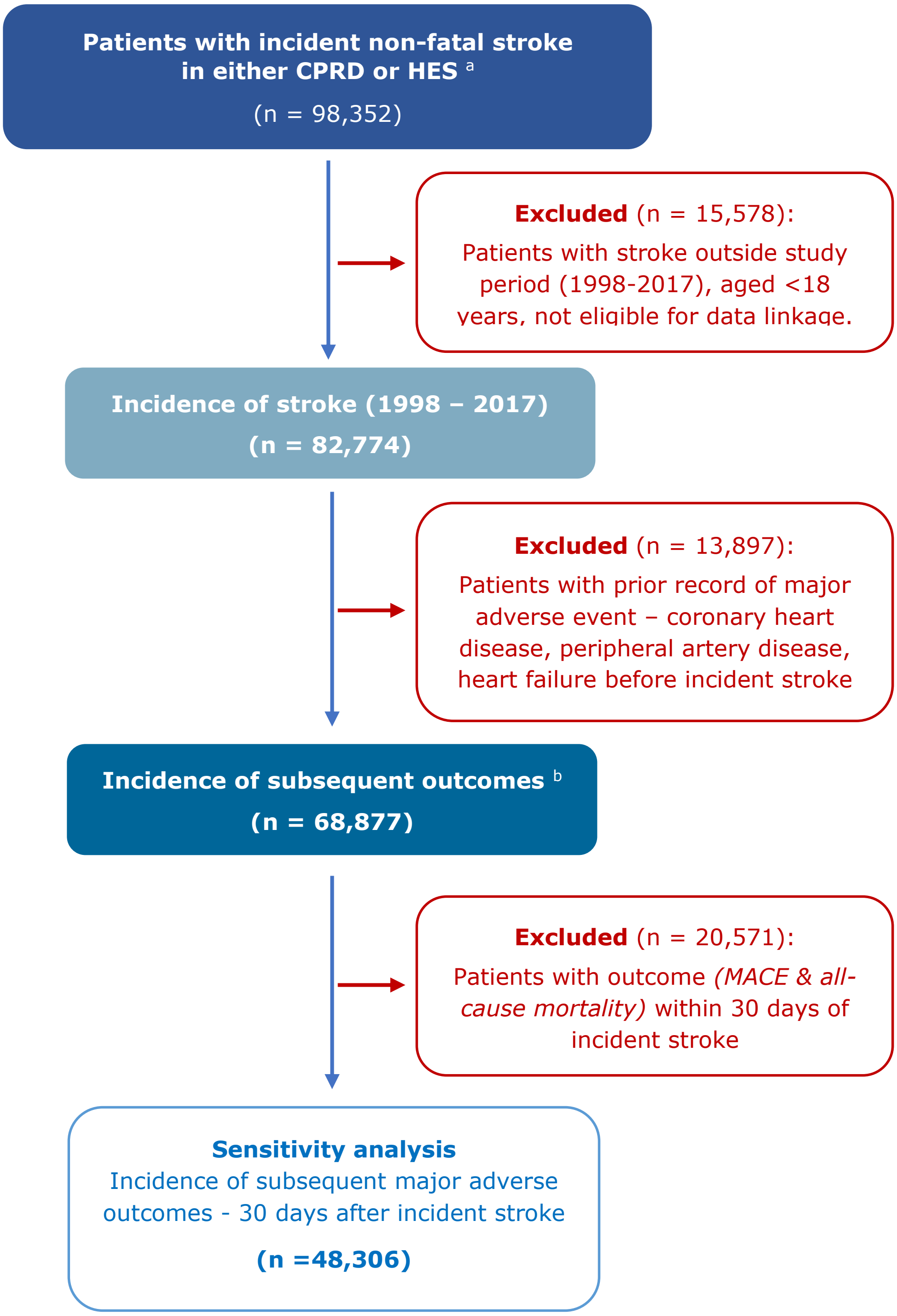

a. Incident non-fatal stroke recorded between 1st January 1998 and 31st December 2017 in patients, 18 years and over with at least 12 months of registration with the practice. Practices contributing up-to-standard data and patient primary care record has linkage to HES.

b. Censored at earliest of outcome of interest, death, transfer out of practice or data end date (last data collection).

Outcome of interest: Major adverse cardiovascular disease (defined as either a diagnosis of coronary heart disease, recurrent stroke, peripheral vascular disease, heart failure and cardiovascular-related death) and all-cause mortality.

Supplemental Figure II **Distribution of subsequent major adverse outcomes by sex and 5-year age group for patient with subsequent outcome after 30 days of incident stroke** (n=48,306)

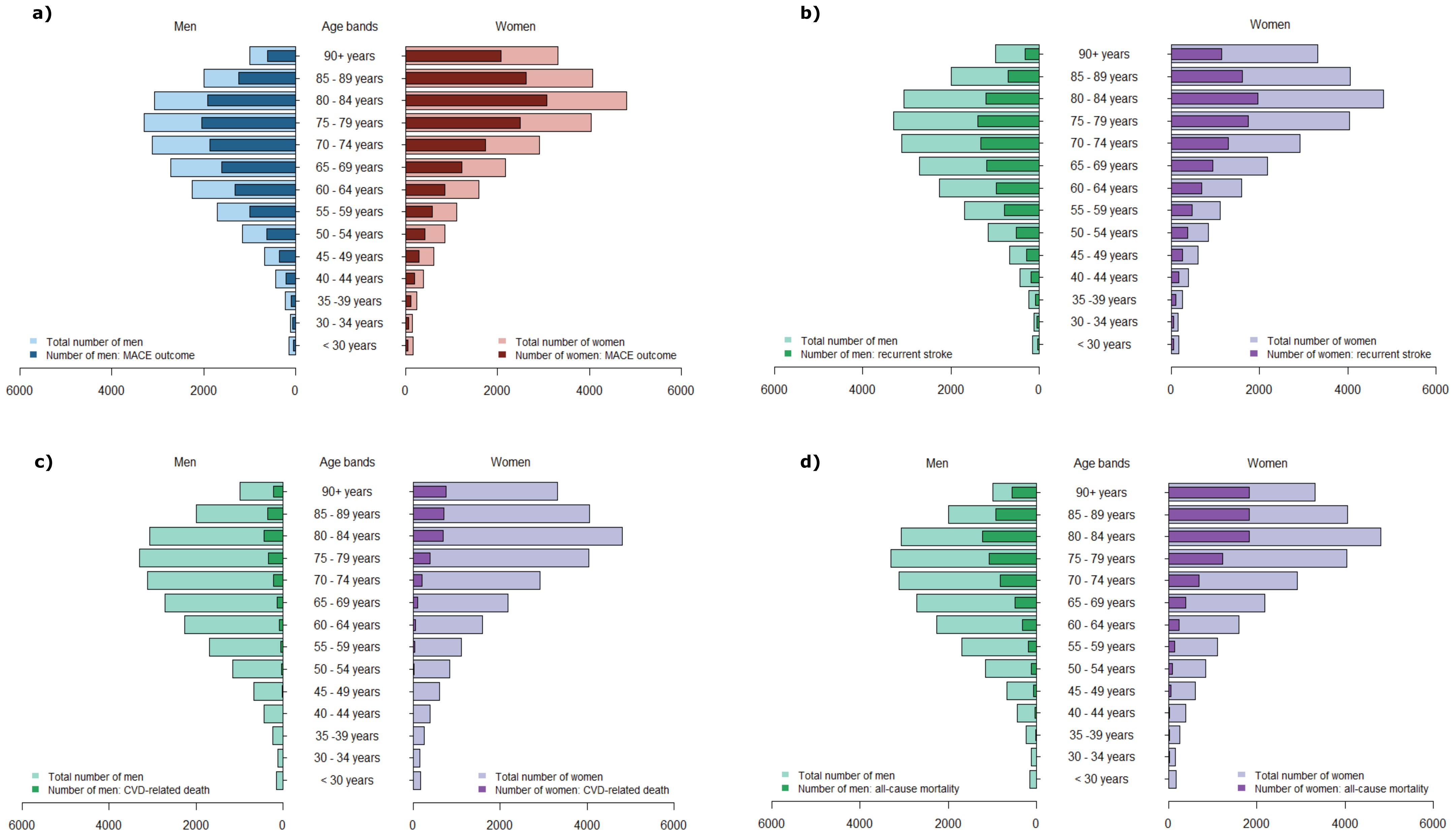

(a) Major adverse cardiovascular events (n=28,750), (b) Recurrent stroke (n=19,896), (c) Cardiovascular-related mortality (n=4,797), (d) All-cause mortality (n=14,137)

Supplemental Figure III **Incidence of subsequent major adverse outcomes presented by sex and 5-year age groups for patient with subsequent major adverse event after 30 days of index stroke** (n=48,306)

a. Major adverse cardiovascular events

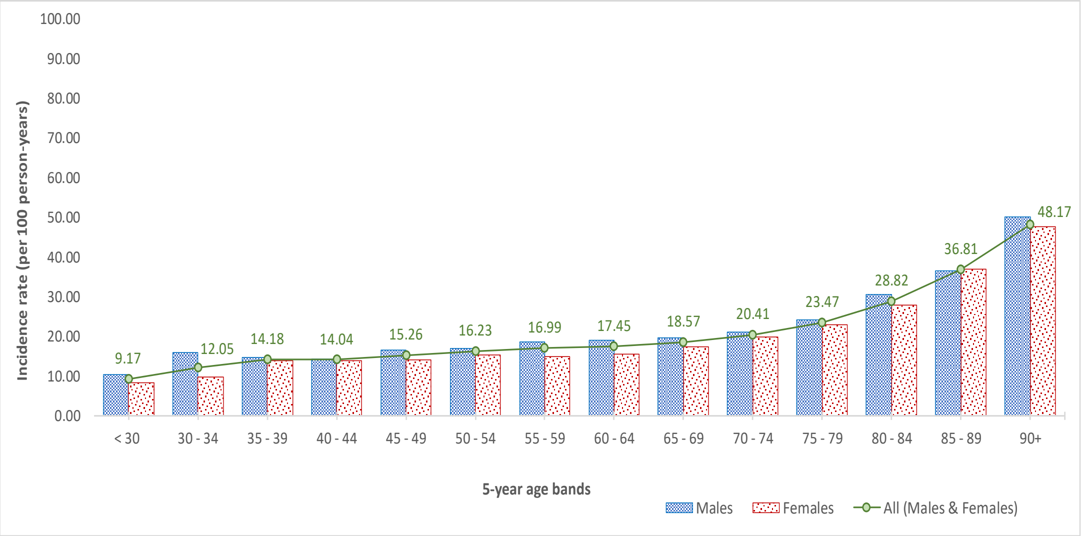

b. Recurrent stroke

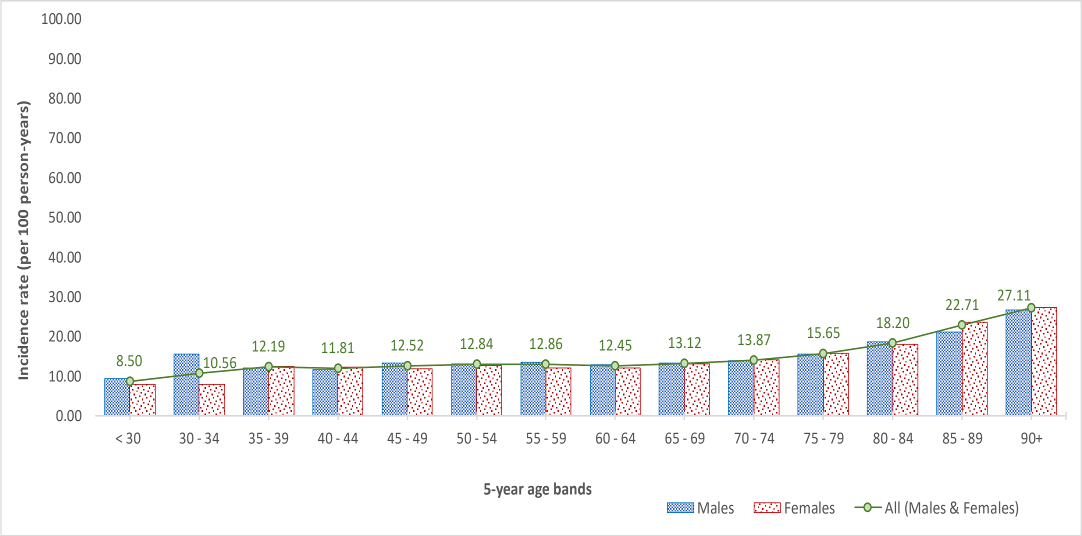

c. Cardiovascular-related mortality

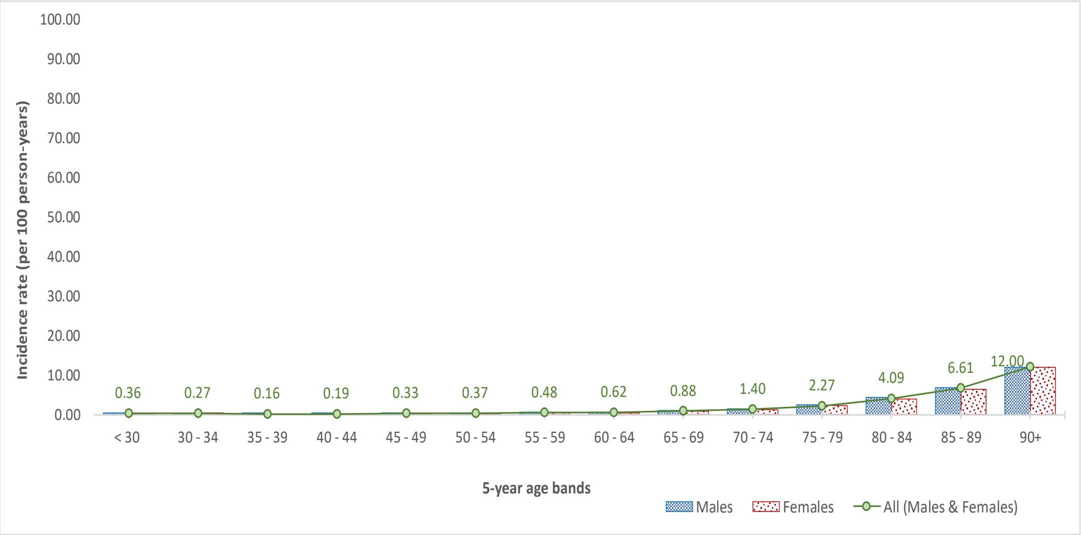

d. All-cause mortality

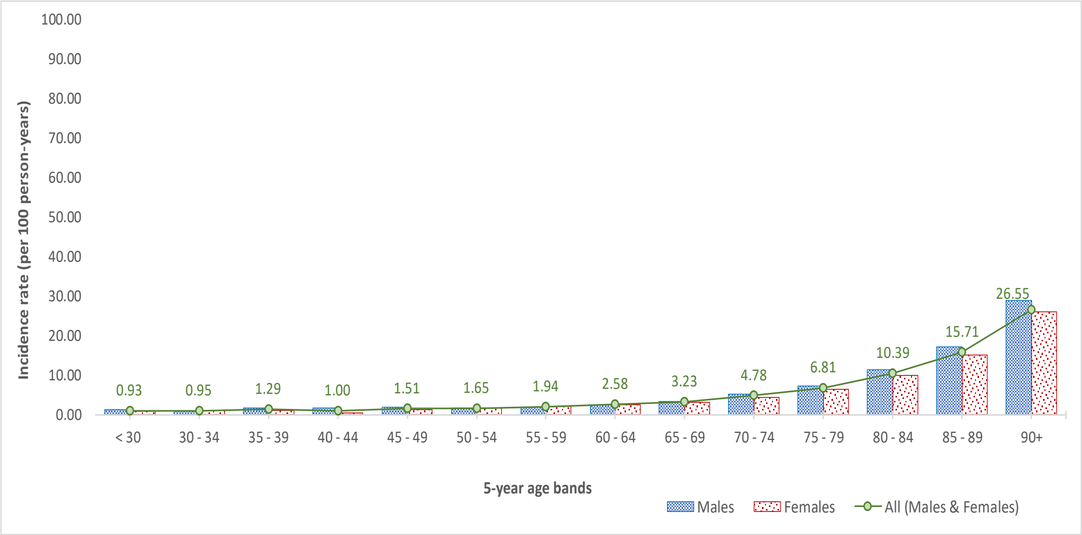

Supplemental Table I ICD-10 and Read code lists for stroke

Stroke subtype	ICD-10 codes	CPRD Read codes
Haemorrhagic stroke	I61, I62.9, I69.1, I69.2	662o.00, 7004300, G610.00, G611.00, G612.00, G613.00, G614.00, G615.00, G616.00, G617.00, G618.00, G619.00, G61..00, G61..11, G61..12, G61X000, G61X100, G61X.00, G61z.00, G681.00, G682.00, Gyu6200, Gyu6F00
Ischaemic stroke	I63.0, I63.4, I63.9, I63.1, I63.5, I69.3, I63.2, I63.6, I63.3, I63.8	G63..11, G63y000, G63y100, G640000, G640.00, G641000, G641.00, G641.11, G64..00, G64..11, G64..12, G64..13, G64z000, G64z100, G64z111, G64z200, G64z300, G64z400, G64z.00, G64z.11, G64z.12, G683.00, G6W..00, G6X..00, Gyu6300, Gyu6400, Gyu6G00
Stroke not-otherwise-specified (NOS)	I64, I69.4, I69.8, G46.3, G46.7, G46.6, G46.5, G46.4, G46.8	14A7.00, 14A7.11, 14A7.12, 14AK.00, 1M4..00, 661M700, 661N700, 662e.00, 662e.11, 662M100, 662M200, 662M.00, 7P24200, 8HHM.00, 8IEC.00, 9h21.00, 9h22.00, 9h2..00, Fyu5600, G663.00, G664.00, G665.00, G666.00, G667.00, G668.00, G66..00, G66..11, G66..12, G66..13, G68X.00, Gyu6C00, L440.11, L440.12, ZV12511, ZV12512

Supplemental Table II **Incidence of stroke presented by year and sex (1998 – 2017)**

	**Stroke events**		**100,000 person-years at risk**		**Incidence rate per 100,000 person-years** (95% CI)		
**Age group** (years)	Men	Women	Men	Women	All	Men	Women
1998	833	1044	7.19	7.58	127.05 (121.43 – 132.93)	115.82 (108.22 – 123.96)	137.71 (129.61 – 146.32)
1999	975	1264	8.21	8.62	132.98 (127.52 – 138.60)	118.68 (111.46 – 126.37)	146.60 (138.73 – 154.91)
2000	1249	1472	10.04	10.49	132.53 (127.64 – 137.60)	124.40 (117.69 – 131.50)	140.30 (133.31 – 147.65)
2001	1564	1942	12.41	12.93	138.30 (133.80 – 142.96)	126.00 (119.91 – 132.40)	150.11 (143.58 – 155.64)
2002	1832	2171	14.01	14.55	140.15 (135.88 – 144.56)	130.73 (124.88 – 136.86)	149.23 (143.08 – 155.71)
2003	2051	2461	16.13	16.70	137.47 (133.51 – 141.54)	127.19 (121.80 – 132.81)	147.40 (141.69 – 153.34)
2004	2732	3510	17.90	18.45	171.72 (167.51 – 176.03)	152.63 (147.02 – 158.47)	190.23 (184.04 – 196.63)
2005	2598	3456	19.39	19.97	153.81 (149.98 – 157.73)	133.95 (128.90 – 139.21)	173.09 (167.42 – 178.96)
2006	2568	3127	19.94	20.50	140.84 (137.23 – 144.54)	128.79 (123.90 – 133.86)	152.56 (147.31 – 158.00)
2007	2415	2823	20.18	20.68	128.19 (124.77 – 131.71)	119.67 (114.99 – 124.54)	136.51 (131.56 – 141.64)
2008	2354	2773	20.46	20.94	123.85 (120.51 – 127.29)	115.07 (110.51 – 119.81)	132.43 (127.59 – 137.45)
2009	2493	2884	20.56	21.05	129.20 (125.79 – 132.70)	121.23 (116.56 – 126.08)	136.98 (132.07 – 142.07)
2010	2515	2793	20.39	20.88	128.62 (125.21 – 132.13)	123.36 (118.64 – 128.28)	133.75 (128.88 – 138.80)
2011	2430	2801	19.96	20.50	129.27 (125.81 – 132.82)	121.71 (116.97 – 126.65)	136.62 (131.65 – 141.77)
2012	2331	2445	19.81	20.37	118.86 (115.54 – 122.28)	117.65 (112.97 – 122.53)	120.04 (115.38 – 124.90)
2013	2215	2422	18.98	19.56	120.33 (116.91 – 123.84)	116.72 (111.96 – 121.68)	123.82 (118.99 – 128.85)
2014	1897	2021	17.54	18.05	110.09 (106.69 – 113.59)	108.18 (103.42 – 113.16)	111.94 (107.16 – 116.93)
2015	1394	1495	15.55	16.01	91.52 (88.24 – 94.92)	89.62 (85.04 – 94.46)	93.36 (88.74 – 98.21)
2016	1023	1020	13.09	13.46	76.96 (73.70 – 80.37)	78.18 (73.53 – 83.12)	75.78 (71.27 – 80.58)
2017	691	690	11.64	11.93	58.58 (55.58 – 61.76)	59.37 (55.10 – 63.97)	57.82 (53.66 – 62.30)

Supplemental Table III **Stroke incidence presented by age group and sex (1998 – 2017)**

	Stroke events		100,000 person-years at risk		Incidence rate per 100,000 person-years (95% CI)		
**Age group** (years)	Men	Women	Men	Women	All	Men	Women
< 20	148	115	38.67	30.29	3.81 (3.38 – 4.30)	3.83 (3.26 – 4.50)	3.80 (3.16 – 4.56)
20 – 24	126	158	26.84	28.30	5.15 (4.58 – 5.79)	4.69 (3.94 – 5.59)	5.58 (4.78 – 6.52)
25 – 29	314	345	34.16	36.62	9.31 (8.63 – 10.05)	9.19 (8.23 – 10.27)	9.42 (8.48 – 10.47)
30 – 34	637	556	40.52	41.44	14.55 (13.75 – 15.40)	15.72 (14.54 – 16.99)	13.42 (12.35 – 14.58)
35 – 39	1126	858	41.22	40.13	24.39 (23.34 – 25.48)	27.31 (25.76 – 28.96)	21.38 (19.99 – 22.86)
40 – 44	1761	1155	38.77	36.70	38.64 (37.26 – 40.07)	45.42 (43.35 – 47.60)	31.48 (29.71 – 33.34)
45 – 49	2582	1629	34.07	32.68	63.09 (61.21 – 65.02)	75.79 (72.93 – 78.77)	49.84 (47.48 – 52.32)
50 – 54	3531	2345	30.27	29.83	97.78 (95.31 – 100.31)	116.66 (112.88 – 120.57)	78.62 (75.50 – 81.87)
55 – 59	4313	3299	25.96	26.67	144.63 (141.42 – 147.92)	166.12 (161.24 – 171.15)	123.71 (199.56 – 128.01)
60 – 64	4958	4453	20.90	22.96	214.56 (210.27 – 218.94)	237.21 (230.70 – 243.90)	193.94 (188.32 – 199.72)
65 – 69	5486	5864	16.85	19.78	309.88 (304.23 – 315.63)	325.63 (317.13 – 334.36)	296.47 (288.97 – 304.15)
70 – 74	5216	6784	11.86	15.75	434.71 (427.00 – 442.56)	439.87 (428.09 – 451.97)	430.82 (420.69 – 441.20)
75 – 79	4092	6807	7.25	11.47	582.02 (571.20 – 593.05)	564.04 (547.02 – 581.59)	593.39 (579.46 – 607.66)
80 – 84	2397	5262	3.59	7.15	713.37 (697.57 – 729.52)	668.01 (641.80 – 695.30)	736.14 (716.51 – 756.30)
85 – 89	1085	3182	1.37	3.59	860.57 (835.14 – 886.79)	790.09 (744.45 – 838.53)	887.57 (857.26 – 918.96)
90 – 94	329	1456	0.39	1.38	1000.00 (959.80 – 1100.00)	838.94 (753.01 – 934.67)	1100.00 (999.86 – 1100.00)
95+	59	346	0.11	0.41	783.57 (710.85 – 863.72)	548.94 (425.31 – 708.50)	845.16 (760.64 – 939.08)
All ages	38,160	44,614	372.80	385.14	109.21 (108.47 – 109.96)	102.36 (101.34 – 103.39)	115.84 (114.77 – 116.92)

Supplemental Table IV **Age- and sex-adjusted incidence rate ratio of stroke, by socioeconomic status**

**Socioeconomic status**	Incident stroke
1 (least deprived)	Reference
2	1.03 (1.01–1.05)
3	1.04 (1.01–1.06)
4	1.09 (1.06–1.11)
5 (most deprived)	1.10 (1.08–1.13)

Supplemental Table V **Demographic characteristics of individuals aged 18 years or above with incident non-fatal stroke and no prior history of major adverse event** (n = 68,877)

	Total<br>n = 68,877	Men<br>n = 31,482 (45.7%)	Women<br>n = 37,395 (54.3%)	*p*-value
**Age at incident stroke**	73.3 (13.9)	70.3 (13.4)	75.9 (13.9)	0.0001
**Type of incident stroke**				<0.001
Haemorrhagic	6,682 (9.7)	3,229 (10.3)	3,453 (9.2)	
Ischaemic	26,146 (38.0)	12,391 (39.4)	13,755 (36.8)	
Not otherwise specified	36,049 (52.3)	15,862 (50.4)	20,187 (54.0)	
**Socioeconomic status**				0.282
1 (Highest SES)	14,779 (21.5)	6,840 (21.7)	7,939 (21.2)	
2	15,350 (22.3)	6,934 (22.0)	8,416 (22.5)	
3	14,870 (21.6)	6,782 (21.5)	8,088 (21.6)	
4	12,661 (18.4)	5,748 (18.3)	6,913 (18.5)	
5 (Lowest SES)	11,101 (16.1)	5,119 (16.3)	5,982 (16.0)	
Missing	116 (0.2)	59 (0.2)	57 (0.2)	
**Ethnicity**				<0.001
Asian	895 (1.3)	479 (1.5)	416 (1.1)	
Black	560 (0.8)	278 (0.9)	282 (0.8)	
Mixed	102 (0.2)	57 (0.2)	45 (0.1)	
Other	481 (0.7)	243 (0.8)	238 (0.6)	
White	61,145 (88.8)	28,070 (89.2)	33,075 (88.5)	
Unknown	5,694 (8.3)	2,355 (7.5)	3,339 (8.9)	
**Comorbid conditions**				
Atrial fibrillation	6,456 (9.4)	2,746 (8.7)	3,710 (9.9)	<0.001
Diabetes mellitus	7,979 (11.6)	3,968 (12.6)	4,011 (10.7)	<0.001
Dyslipidaemia	6,562 (9.5)	2,809 (8.9)	3,753 (10.0)	<0.001
Hypertension	31,861 (46.3)	13,388 (42.5)	18,473 (49.4)	<0.001
TIA	14,073 (20.4)	6,257 (19.9)	7,816 (20.9)	0.001
**Major adverse outcomes**				<0.001
Coronary heart disease	2,420 (4.1)	1,311 (5.0)	1,109 (3.5)	
Haemorrhagic stroke	2,378 (4.1)	1,209 (4.6)	1,169 (3.6)	
Ischaemic stroke	8,842 (15.1)	4,254 (16.1)	4,588 (14.3)	
Stroke (not specified)	22,611 (38.6)	10,512 (39.7)	12,099 (37.6)	
Peripheral vascular disease	593 (1.0)	334 (1.3)	259 (0.8)	
Heart failure	1,482 (2.5)	633 (2.4)	849 (2.6)	
CVD-related death	9,174 (15.6)	3,516 (13.3)	5,658 (17.6)	
Non-CVD related death	11,161 (19.0)	4,734 (17.9)	6,427 (20.0)	

TIA: transient ischaemic attack; n: total number; %: percentage/proportion; Mean age at incident stroke reported with standard deviation.

Major adverse event defined as a record of either coronary heart disease, peripheral vascular disease or heart failure.

Supplemental Table VI **Age- and sex-adjusted incidence rate ratio of subsequent major adverse outcomes, by socioeconomic status**

Socioeconomic status	MACE	Recurrent stroke	CVD-related mortality	All-cause mortality
1 (Highest SES)	Reference	Reference	Reference	Reference
2	1.04 (1.01 – 1.07)	1.04 (1.00 – 1.07)	1.10 (1.03 – 1.17)	1.07 (1.02 – 1.11)
3	1.09 (1.06 – 1.12)	1.07 (1.04 – 1.11)	1.07 (1.00 – 1.14)	1.05 (1.00 – 1.09)
4	1.11 (1.08 – 1.14)	1.05 (1.01 – 1.08)	1.21 (1.13 – 1.29)	1.16 (1.11 – 1.21)
5 (Lowest SES)	1.09 (1.06 – 1.13)	1.00 (0.97 – 1.04)	1.31 (1.23 – 1.41)	1.31 (1.26 – 1.37)

Supplemental Table VII **Descriptive characteristics of patients with subsequent outcome within 30 days compared to those with outcomes after 30 days of incident stroke**

	**Outcome within 30 days** n=20,571 (29.9%)	**Outcome after 30 days** n=48,306 (70.1%)	***p*-value**
**Age at incident stroke**	73.8 (14.0)	73.1 (13.9)	0.0001
**Age at subsequent MACE outcome**	73.6 (14.0)	76.3 (13.1)	0.0001
**Female**	10,995 (53.5)	26,400 (54.7)	0.004
**Socioeconomic status**			0.714
1 (Highest SES)	4,457 (21.7)	10,322 (21.4)	
2	4,561 (22.2)	10,789 (22.3)	
3	4,481 (21.8)	10,389 (21.5)	
4	3,783 (18.4)	8,878 (18.4)	
5 (Lowest SES)	3,254 (15.8)	7,847 (16.2)	
Missing	35 (0.2)	81 (0.2)	
**Ethnicity**			<0.001
Asian	280 (1.4)	615 (1.3)	
Black	181 (0.9)	379 (0.8)	
Mixed	29 (0.1)	73 (0.2)	
Other	145 (0.7)	336 (0.7)	
White	17,966 (87.3)	43,179 (83.4)	
Unknown	1,970 (9.6)	3,724 (7.7)	
**Comorbid conditions**			
Atrial fibrillation	2,244 (10.9)	4,212 (8.7)	<0.001
Diabetes mellitus	2,484 (12.1)	5,495 (11.4)	0.009
Dyslipidaemia	1,715 (8.3)	4,847 (10.0)	<0.001
Hypertension	9,400 (45.70)	22,461 (46.5)	0.053
TIA	1,696 (8.2)	12,377 (25.6)	<0.001
**Major adverse outcomes**			<0.001
Coronary heart disease	291 (1.4)	2,129 (5.6)	
Haemorrhagic stroke	1,448 (7.0)	930 (2.44)	
Ischaemic stroke	5,247 (25.5)	3,595 (9.4)	
Stroke (not specified)	7,240 (35.2)	15,371 (40.4)	
Peripheral vascular disease	60 (0.29)	533 (1.4)	
Heart failure	87 (0.42)	1,395 (3.7)	
CVD-related death	4,377 (21.3)	4,797 (12.6)	
Non-CVD related death	1,821 (8.9)	9,340 (24.5)	

n: total number; %: percentage/proportion; Mean age at incident stroke and mean age at subsequent MACE outcome are reported with standard deviation.

Supplemental Table VIII **Descriptive characteristics of patients with subsequent outcome after 30 days of incident stroke by sex**

	**Total**<br>n = 48,306	**Men**<br>n =21,906 (45.4%)	**Women**<br>n =26,400 (54.7%)	***p*-value**
**Age at incident stroke**	73.1 (13.9)	70.3 (13.4)	75.5 (13.9)	0.0001
**Age at subsequent MACE outcome**	76.3 (13.1)	73.3 (12.8)	78.8 (12.9)	0.0001
**Socioeconomic status**				0.646
1 (Highest SES)	10,322 (21.4)	4,730 (21.6)	5,957 (22.6)	
2	10,789 (22.3)	4,832 (22.1)	5,957 (22.6)	
3	10,389 (21.5)	4,691 (21.4)	5,698 (21.6)	
4	8,878 (18.4)	4,018 (18.3)	4,860 (18.4)	
5 (Lowest SES)	7,847 (16.2)	3,597 (16.4)	4,250 (16.1)	
Missing	81 (0.2)	38 (0.2)	43 (0.2)	
**Ethnicity**				<0.001
Asian	615 (1.3)	327 (1.5)	288 (1.1)	
Black	379 (0.8)	183 (0.8)	196 (0.7)	
Mixed	73 (0.2)	38 (0.2)	35 (0.1)	
Other	336 (0.7)	163 (0.7)	173 (0.7)	
White	43,179 (89.4)	19,644 (89.7)	23,535 (89.2)	
Unknown	3,724 (7.7)	1,551 (7.1)	2,173 (8.2)	
**Comorbid conditions**				
Atrial fibrillation	4,212 (8.7)	1,839 (8.4)	2,373 (9.0)	0.021
Diabetes mellitus	5, 495 (11.4)	2,739 (12.5)	2,756 (10.4)	<0.001
Dyslipidaemia	4,847 (10.0)	2,083 (9.5)	2,764 (10.5)	<0.001
Hypertension	22,461 (46.5)	9,402 (42.9)	13,059 (49.5)	<0.001
TIA	12,377 (25.6)	5,497 (25.1)	6,880 (26.1)	0.015
**Outcomes**				<0.001
Coronary heart disease	2,129 (4.4)	1,149 (5.2)	980 (3.7)	
Haemorrhagic stroke	930 (1.9)	463 (2.1)	467 (1.8)	
Ischaemic stroke	3,595 (7.4)	1,641 (7.5)	1,954 (7.4)	
Stroke (not specified)	15,371 (31.8)	6,944 (31.7)	8,427 (31.9)	
Peripheral vascular disease	533 (1.1)	301 (1.4)	232 (0.9)	
Heart failure	1,395 (2.9)	593 (2.7)	802 (3.0)	
CVD-related death	4,797 (9.9)	1,882 (8.6)	2,915 (11.0)	
Non-CVD related death	9,340 (19.3)	3,954 (18.0)	5,386 (20.4)	

n: total number; %: percentage/proportion; Mean age at incident stroke and mean age at subsequent MACE outcome reported with standard deviation.

Supplemental Table IX **Incidence of subsequent major adverse outcomes for patient with subsequent major adverse event after 30 days of index stroke** (n=48,306)

	Follow-up time	Cases	Person-years*	Incidence rate (per 100 person-years)	Incidence rate ratio	*p*-value
**MACE** (All)	1.09 (0.31 – 2.91)	28,750	1,200	23.14 (22.87 – 23.41)		<0.0001
Men	1.09 (0.33 – 2.98)	12,973	575.95	22.53 (22.14 – 22.92)	Reference	
Women	1.09 (0.31 – 2.85)	15,777	666.62	23.67 923.30 – 24.04)	1.05 (1.03 – 1.08)	
**Recurrent stroke** (All)	1.00 (0.28 – 2.29)	19,896	1,300	15.43 (15.22 – 15.65)		<0.0001
Men	0.99 (0.28 – 2.32)	9,048	611.13	14.81 (14.50 – 15.11)	Reference	
Women	1.02 (0.29 – 2.28)	10,848	678.38	15.99 (15.69 – 16.30)	1.08 (1.05 – 1.11)	
**Cardiovascular mortality** (All)	1.33 (0.25 – 3.85)	4,797	2,100	2.34 (2.27 – 2.41)		<0.0001
Men	1.55 (0.33 – 4.29)	1,882	971.16	1.94 (1.85 – 2.03)	Reference	
Women	1.16 (0.23 – 3.62)	2,915	1,100	2.70 (2.60 – 2.80)	1.39 (1.31 – 1.48)	
**All-cause mortality** (All)	1.83 (0.44 – 4.66)	14,137	2,100	6.58 (6.47 – 6.69)		<0.0001
Men	1.90 (0.49 – 4.78)	5,836	1,000	5.78 (5.64 – 5.93)	Reference	
Women	1.83 (0.44 – 4.66)	8,301	1,100	7.28 (7.12 – 7.44)	1.26 (1.22 – 1.30)	
**Coronary heart disease** (All)	2.18 (0.82 – 4.63)	2,129	2,000	1.09 (1.05 – 1.14)		<0.0001
Men	2.17 (0.78 – 4.68)	1,149	921.94	1.25 (1.18 – 1.32)	Reference	
Women	2.18 (0.86 – 4.41)	980	1000	0.95 (0.89 – 1.01)	0.76 (0.70 – 0.83)	
**Peripheral arterial disease** (All)	1.88 (0.82 – 4.30)	533	2,000	0.27 (0.25 – 0.29)		<0.0001
Men	1.99 (0.81 – 4.21)	301	945.69	0.32 (0.28 – 0.36)	Reference	
Women	1.80 (0.84 – 4.38)	232	1,000	0.22 (0.19 – 0.25)	0.70 (0.58 – 0.83)	
**Heart failure** (All)	1.94 (0.69 – 4.68)	1,395	2,000	0.70 (0.67 – 0.74)		0.0001
Men	1.91 (0.70 – 4.52)	593	946.69	0.63 (0.58 – 0.68)	Reference	
Women	1.95 (0.69 – 4.90)	802	1,000	0.77 (0.72 – 0.82)	1.23 (1.10 – 1.37)	

* 100 person-years at risk; All – both men and women; Follow-up time – median follow-up time in years reported with interquartile range

Supplemental Table X **Age- and sex-adjusted incidence rate ratio of subsequent major adverse outcomes, by socioeconomic status for patient with subsequent major adverse event after 30 days of index stroke** (n=48,306)

Socioeconomic status	MACE	Recurrent stroke	CVD-related mortality	All-cause mortality
1 (Highest SES)	Reference	Reference	Reference	Reference
2	1.06 (1.02 – 1.10)	1.06 (1.01 – 1.04)	1.06 (0.97 – 1.15)	1.03 (0.98 – 1.08)
3	1.10 (1.06 – 1.14)	1.08 (1.03 – 1.13)	1.07 (0.98 – 1.17)	1.04 (0.99 – 1.09)
4	1.13 (1.09 – 1.17)	1.08 (1.03 – 1.12)	1.15 (1.05 – 1.26)	1.12 (1.07 – 1.18)
5 (Lowest SES)	1.13 (1.09 – 1.18)	1.03 (0.99 – 1.08)	1.32 (1.21 – 1.45)	1.30 (1.23 – 1.37)
