# Supplementary figures and images for: Sex, Age, and Socioeconomic Differences in Nonfatal Stroke Incidence and Subsequent Major Adverse Outcomes
Source: Stroke. 2021 Jan 26;52(2):396–405. doi: 10.1161/STROKEAHA.120.031659 (PMC7834661; doi:10.1161/STROKEAHA.120.031659)

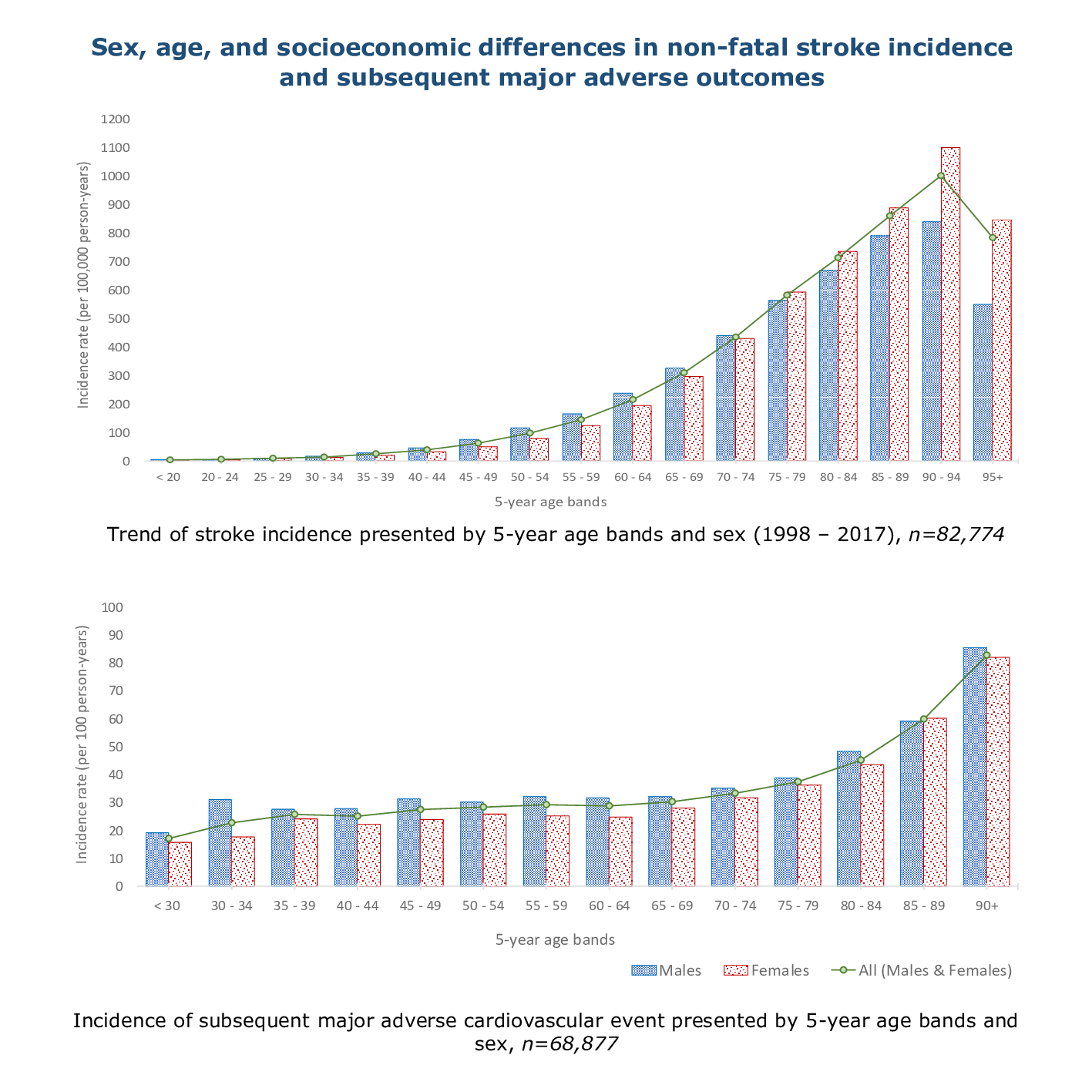

Supplement: Supplementary file 2 [file str-52-396-s002.jpg]
